# Acceptability of Primary Care Counseling and Brief Educational Messages to Increase Awareness about Alcohol and Breast Cancer Risks among Bisexual and Lesbian Women

**DOI:** 10.3390/ijerph20054184

**Published:** 2023-02-26

**Authors:** Adelaide Balenger, Lia C. Scott, Monica H. Swahn, Ritu Aneja

**Affiliations:** 1 School of Public Health, Georgia State University, Atlanta, GA 30302, USA; 2 Wellstar College of Health and Human Services, Kennesaw State University, Kennesaw, GA 30144, USA; 3 School of Health Professions, The University of Alabama at Birmingham, Birmingham, AL 35294, USA

**Keywords:** alcohol, substance use, bisexual, lesbian, sexual minority women, breast cancer

## Abstract

This research had two aims: (1) to assess how often bisexual and lesbian women self-report screening and counseling for alcohol use in primary care settings; and (2) understand how bisexual and lesbian women respond to brief messages that alcohol increases breast cancer risk. The study sample consisted of 4891 adult U.S. women who responded to an online, cross-sectional Qualtrics survey in September–October 2021. The survey included the Alcohol Use Disorders Identification Test (AUDIT), questions about alcohol screening and brief counseling in primary care, and questions assessing awareness of the link between alcohol use and breast cancer. Bivariate analyses and logistic regression were conducted. Bisexual and lesbian women had higher odds of harmful drinking (AUDIT score ≥ 8) than heterosexual women (adjusted odds ratio [AOR] = 1.26, 95% confidence interval [CI] = 1.01–1.57 for bisexual women; AOR =1.78, 95% CI = 1.24–2.57 for lesbian women). However, bisexual and lesbian women were no more likely than heterosexual women to be advised about drinking in primary care. In addition, bisexual, lesbian, and heterosexual women had similar reactions to messages highlighting that alcohol is a risk factor for breast cancer. Women across all three sexual orientations who are harmful drinkers more often agreed to search for more information online or talk to a medical professional compared to non-harmful drinkers.

## 1. Introduction

Alcohol use is associated with a myriad of diseases and injuries, including many cancers [1,2,3]. Nevertheless, alcohol use and binge drinking among adults in the U.S. are increasing, particularly among adult women [4]. This trend calls for greater attention to the unique risks that alcohol poses to women, including breast cancer. Alcohol consumption increases the risk of breast cancer with a dose–response relationship, meaning even light or moderate drinking increases breast cancer risk [5,6,7,8]. However, among a representative sample of U.S. women aged 15–44, three in four women were unaware of this link [9]. Other studies in the U.S., England, and Denmark have found that more people are aware of the link between alcohol use and other cancers, including liver cancer and esophageal cancer, compared to breast cancer [10,11,12,13].

While all women need to learn about the association between alcohol use and breast cancer, bisexual and lesbian women often drink more frequently, engage in riskier drinking patterns, experience more alcohol-related consequences, and are diagnosed with alcohol use disorders at higher rates compared to heterosexual women [14,15,16,17,18,19,20]. As a result, bisexual and lesbian women may be at an increased risk for breast cancer, although more research is needed on this topic [21]. Previous research has pointed to the sexual minority stress model, connecting the stigma, prejudice, and discrimination experienced by sexual minorities as the reason for the increased substance use [14,22,23]. Most studies on alcohol use among sexual minority populations have focused on interventions for heavy alcohol consumption, predominantly among sexual minority men [24]. Scarce research has assessed interventions to increase awareness of the long-term harms of alcohol among bisexual and lesbian women.

Screening and counseling for alcohol use in primary care settings are recommended for all adults by the U.S. Preventive Services Task Force and provide a chance for health care professionals to inform patients about both the short-term and long-term effects of alcohol use [25]. In 2017, 81.8% of female U.S. adults reported being asked about alcohol use at a checkup in the last two years. Among those who reported binge drinking, only 33.8% were advised about the harms associated with excessive drinking, and only 13.7% were advised to reduce or quit drinking [26]. Yet, the researchers did not disaggregate these results by sexual orientation. Furthermore, sexual minority women are less likely to access usual places of care than their heterosexual peers, and health care professionals are often inadequately trained on the unique needs of sexual minority women [15,27]. Thus, alcohol use screening and brief counseling in primary care may require additional considerations for sexual minority women.

Health education messages, including mass media campaigns, are another opportunity to increase awareness of the risk of cancer from alcohol. Studies in Denmark, England, and Australia have shown that mass media campaigns, via social media and television, can increase knowledge about the association between alcohol and cancer [12,28,29]. Similarly, social media campaigns have been shown to increase knowledge about the harms of cigarettes and improve cessation efforts [30,31,32]. These cost-effective strategies and best practices for tobacco prevention could be adapted for alcohol behavior modifications. However, no research to date has examined their ability to increase awareness about the risks of drinking among bisexual and lesbian women.

## 2. Objective

The purpose of this study was to (1) assess how often bisexual and lesbian women self-report screening and counseling for alcohol use in primary care settings and (2) understand how bisexual and lesbian women respond to brief health educational messages highlighting that alcohol increases breast cancer risk. Both brief educational interventions represent two opportunities to educate bisexual and lesbian women on the long-term health risks of drinking.

## 3. Materials & Methods

### 3.1. Study Sample and Survey Distribution

The study sample consisted of adult U.S. women who responded to an anonymous, internet-based Qualtrics survey, the Alcohol and Breast Cancer Link AwarEness (ABLE) survey. Respondents provided informed consent at the start of the survey. Online samples recruited by Qualtrics typically include individuals who agreed to be contacted by a market research service [33]. Since Qualtrics handled participant recruitment and distribution of the survey, no response rate could be computed. Women received compensation for the survey based on Qualtrics’ discretion. Previous research has recognized the value of online samples in other populations due to their efficiency in gathering epidemiologic data [34,35].

Eligibility criteria included residence in the U.S., ≥18 years of age, and identification as female. We provided Qualtrics the following quotas for the online sample: for age, 20% aged 18–24, 20% aged 25–34; 20% aged 35–44; 20% aged 45–54, and 20% aged 55+; for race, at least 15% Black/African American; for ethnicity, at least 10% Spanish/Hispanic/Latino origin. Although all fifty U.S. states and Washington D.C. are represented in the survey, 21 states represented about 80% of the survey respondents. Qualtrics distributed the online survey from September 16, 2021 to October 14, 2021. The study was approved by the Georgia State University Institutional Review Board (H21673).

### 3.2. Measures

#### 3.2.1. Sociodemographic Characteristics

The survey included questions on demographic and socioeconomic variables, including sexual orientation, age, race/ethnicity, health insurance status, marital status, education, income, and family history of drug or alcohol use disorder. For sexual orientation, respondents chose from heterosexual or straight, gay or lesbian, bisexual, other, or prefer not to say.

#### 3.2.2. Harmful Drinking

We used the well-recognized and validated Alcohol Use Disorders Identification Test (AUDIT) to assess the level of alcohol consumption [36]. A cut-off score of ≥8 was considered harmful or hazardous drinking, as recommended by the World Health Organization [36]. 

#### 3.2.3. Alcohol Screening and Brief Counseling in Primary Care Settings

Questions about how often primary care professionals ask alcohol-related questions were taken from the 2019 Behavioral Risk Factor Surveillance System (Module 24) [37]. The first question was how long it had been since their last routine check-up. If the respondent answered that their checkup was within the last one or two years, they indicated whether they were asked at that checkup if they drank alcohol and how much. If affirmative, another question followed about if they were specifically asked about binge drinking (consuming four or more drinks on at least one occasion in the last 30 days). If they answered that their check-up was within the last one or two years, two questions asked if they were advised about a harmful level of alcohol use or to reduce/quit their drinking [37].

#### 3.2.4. Belief That Alcohol Increases Breast Cancer Risk and Reactions to This Information

**Figure 1:**
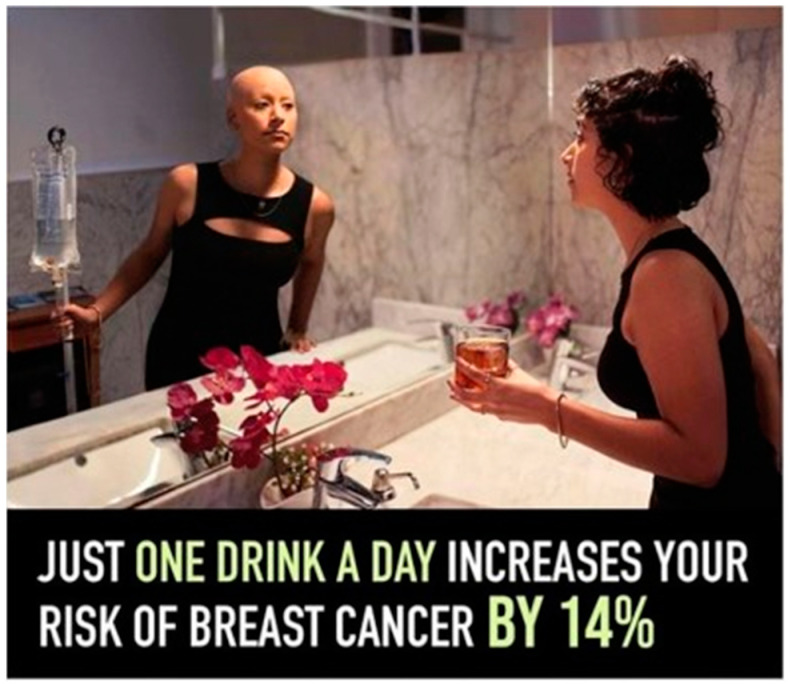
Drink Less for Your Breasts [38] message on the ABLE Survey. Consent provided by Priscilla Martinez to include this image in the survey.

**Figure 2:**
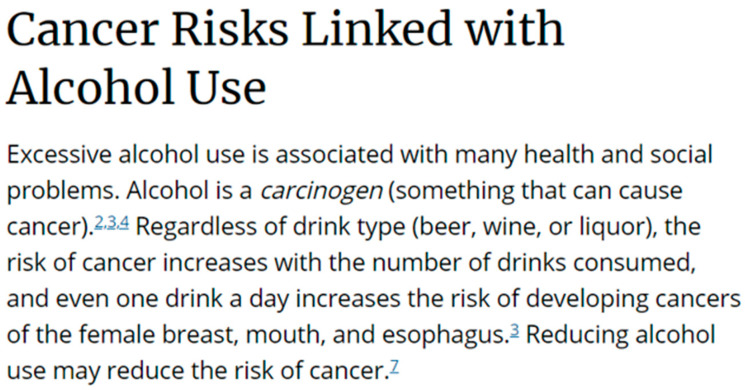
CDC Website Excerpt [39] message on the ABLE Survey. Citations embedded in the website excerpt were not listed in the survey.

Women were asked the following: “Do you think your risk of developing the following types of cancer is increased by drinking alcohol?”, where women indicated yes, no, or do not know for eight types of cancer, including breast cancer [12]. Subsequently, respondents were randomized to see one of three messages—(1) an Instagram post from the “Drink Less for Your Breasts” campaign that says, “Just one drink a day can increase your risk of breast cancer by 14%”; (2) an excerpt from the CDC website on the association between alcohol and cancers; or (3) both the Instagram post and the CDC website excerpt (Figure 1 and Figure 2) [38,39].

After the survey educational messages, participants were asked again whether they thought their risk of developing breast cancer was increased by drinking alcohol. Respondents then indicated their level of agreement on a Likert scale with their intention to search for more information online or to talk to a medical professional about the link between alcohol and breast cancer. These intentions were loosely adapted from another study assessing intentions related to HPV vaccinations after seeing messages on Facebook [40,41].

### 3.3. Statistical Analyses

Among the 5027 women who responded to the survey, 136 women were excluded for not disclosing their sexual orientation or identifying as other, so the analysis dataset consisted of 4891 women who identified as bisexual, lesbian, or heterosexual. The research team conducted bivariate analyses using Chi-square tests to detect statistically significant differences for sociodemographic characteristics, harmful/hazardous drinking, beliefs pre- and post- educational messages that alcohol increases breast cancer risk, the prevalence of alcohol screening and brief counseling in primary care, and agreement with intentions to find out more information after viewing the brief educational messages among these three groups. For age category, 26–35 was selected as the reference group since this age category included the largest sample size after the age category 18–25. The age category 18–25 was not selected as the reference group given that the minimum age for legal drinking in the U.S. is 21 [42]. Logistic regression was used to obtain unadjusted and adjusted odds ratios for the outcome harmful drinking and the outcomes related to alcohol screening and brief counseling in primary care. All statistical analyses were performed using SAS version 9.4.

## 4. Results

Among the 4891 women in the analysis dataset, 554 (11.33%) identified as bisexual, 157 (3.21%) as lesbian, and 4180 (85.46%) as heterosexual (Table 1). The age distribution skewed younger among bisexual and lesbian respondents than among heterosexual respondents (Table 1). Compared to heterosexual women, fewer bisexual and lesbian women were married or living with a partner, and more bisexual and lesbian women had a yearly income <$25K (Table 1).

### 4.1. Harmful Drinking and Family History of Alcohol/drug Use Disorder

Both bisexual and lesbian women had higher proportions of harmful drinking, based on an AUDIT score ≥8 (27.08% among bisexual women; 29.94% among lesbian women; 15.91% among heterosexual women) (Table 1). Additionally, bisexual and lesbian women had higher proportions of a family history of drug/alcohol use disorder than heterosexual women (64.86% among bisexual women; 59.24% among lesbian women; 48.47% among heterosexual women) (Table 1).

Both bisexual and lesbian women had higher odds of harmful drinking than heterosexual women, after adjusting for age, race/ethnicity, education, family history of drug/alcohol use disorder, and marital status (adjusted odds ratio [AOR] = 1.26, 95% confidence interval [CI] = 1.01–1.57 for bisexual women; AOR = 1.78; 95% CI = 1.24–2.57 for lesbian women) (Table 2). Black/African American Non-Hispanic women also had a higher odds of harmful drinking (AOR = 1.42; 95% CI = 1.13–1.78) (Table 2). Income was excluded as a co-variate given the missing data. We ran another model with the same variables and an additional interaction term for sexual orientation and race/ethnicity, finding that race/ethnicity did not modify the association between sexual orientation and harmful drinking.

### 4.2. Self-Reported Alcohol Screening and Brief Counseling in Primary Care Setting

A lower proportion of bisexual and lesbian women visited a doctor for a routine checkup within the past year (56.14% among bisexual women; 54.14% among lesbian women; 63.53% among heterosexual women) (Table 1). Bisexual women had a slightly higher proportion of their doctor advising them about a harmful level of drinking (15.42% among bisexual women; 13.91% among lesbian women; 13.48% among heterosexual women) (Table 3).

Logistic regression results indicated that lesbian women had a slightly lower odds of being asked in person or on a form if they drank alcohol (AOR = 0.63; 95% CI = 0.41–0.97) than heterosexual women, after adjusting for age, race/ethnicity, education level, harmful drinking, health insurance status, and a family history of an alcohol/drug use disorder (Table 4). Otherwise, we found no evidence that bisexual or lesbian women were more likely to be advised about alcohol use than heterosexual women. However, women who engaged in harmful drinking had lower odds of visiting a doctor for a routine check-up in the last two years (AOR = 0.81; 95% CI = 0.66–0.99), but higher odds if they were asked how much they drink (AOR = 1.38; 95% CI = 1.05–1.82), if they were asked about binge drinking (AOR = 2.42; 95% CI = 1.92–3.06), if they were offered advice about harmful drinking (AOR = 3.46; CI = 2.78–4.30), and if they were advised to reduce or quit their drinking (AOR = 6.99; 95% CI = 5.47–8.93).

### 4.3. CDC and Drink Less for Your Breasts Brief Educational Messages

Bisexual, lesbian, and heterosexual women had similar levels of awareness that alcohol increases breast cancer risk, even after disaggregating by harmful drinking, both before and after the brief educational messages (Table 5). Among non-harmful drinkers, bisexual women indicated greater agreement with the intention to search for more information online about the link between alcohol and breast cancer (51.12% among bisexual women vs. 43.64% among lesbian women and 43.38% among heterosexual women) (Table 6). Otherwise, no statistically significant differences emerged by sexual orientation related to the intentions.

However, for both intentions (search for more information online about the link between alcohol and breast cancer and talk to a medical professional about the link between alcohol and breast cancer), bisexual, lesbian, and heterosexual women who engaged in harmful drinking indicated greater agreement across the board after the brief educational messages than women who did not report harmful drinking (Table 6). 

## 5. Discussion

Our findings align with previous research that bisexual and lesbian women likely engage in harmful drinking more frequently than their heterosexual peers. Previous studies have shown that bisexual women have the highest risk of harmful drinking [14]. As the confidence intervals for bisexual and lesbian women overlapped in our study, our results are in line with another study that found no significant difference in problematic drinking between lesbian and bisexual women [43]. Regardless, our study reinforces that both bisexual and lesbian women engage in riskier drinking patterns than heterosexual women. In addition, bisexual women, and to a lesser degree lesbian women, had a higher prevalence of a family history of alcohol and substance use disorders, also in line with previous research [17,44]. The reasons for the higher prevalence of a family history of alcohol and substance use disorders are not clear and need to be researched further. Regardless, this finding suggests that sexual minority women may more often have a family history of substance use, another risk factor for harmful drinking [45].

We found that Black women had higher odds of harmful drinking, after adjusting for covariates. Previous studies have shown a higher risk of alcohol and other drug use among sexual minority women of color compared to that among white sexual minority women [14,46]. Although we did not find any evidence for effect modification between sexual orientation and harmful drinking by race/ethnicity, our research was likely limited by the small subgroup of sexual minority women of color. Future studies should examine this interaction with a larger subgroup.

A novel contribution of our study, however, is that both bisexual and lesbian women were no more likely than their heterosexual peers to be offered advice about harmful drinking or advised to quit or reduce drinking by a primary care professional. Notably, the percentage of women asked about alcohol use in primary care settings is lower than what the CDC reported (81.8%) [26]. This is compared to our findings for bisexual (70.98%), lesbian (57.39%), and heterosexual women (67.23%). Primary care providers were more likely to ask about and offer advice to women who reported harmful drinking than to women who did not. Our study confirms that bisexual and lesbian women more often engage in riskier drinking; hence, it is surprising that we found that bisexual and lesbian women were not more likely to be offered advice about drinking in primary care in the unadjusted logistic regression analysis. This may be a result of bisexual and lesbian women comprising only a small subset of the sample.

Despite encouraging findings that primary care providers were more likely to advise women who are harmful drinkers, ideally this brief counseling could also target women who may be at risk for harmful drinking but have not yet started to engage in risky drinking. Women who partake in light or moderate drinking would also benefit from learning about alcohol harms, including the association between alcohol use and breast cancer. Based on our survey, many women indicated that they would talk to a medical professional about the link between alcohol and breast cancer. This willingness to engage with health care providers about the potential long-term harms of alcohol implies the acceptability of screening and counseling for alcohol use in primary care, even among women who do not engage in excessive drinking.

While alcohol screening and brief counseling are promising strategies, health care professionals must be equipped with the knowledge and competencies required for this brief intervention. A qualitative study in the U.K. found that health care professionals often lacked confidence and were fearful of offering incorrect information about alcohol’s link to breast cancer [47]. Given the low awareness that alcohol increases breast cancer risk in the U.S., many health care professionals likely are unaware of this link themselves [9]. In addition, health care professionals frequently lack training to care for sexual minority patients, and sexual minority individuals may not feel comfortable disclosing their sexual orientation to health care providers [27,48]. Given recent evidence among Black sexual minority men and sexual minority youth that disclosure of sexual orientation to health care providers can lead to greater health care utilization, creating welcoming environments for sexual minority patients is needed [49,50].

No significant differences emerged in the reactions to the survey educational messages conveying the association between alcohol use and breast cancer when comparing bisexual, lesbian, and heterosexual women. This finding is perhaps expected, given that both the Drink Less for Your Breasts post and CDC website excerpt were not tailored to a specific sexual orientation. Regarding awareness, the proportion of women who indicated that alcohol is a risk factor for breast cancer increased from about one in four prior to the brief educational messages (in line with previous research) [9] to one in two after seeing the educational messages. Women who were harmful drinkers more frequently indicated that they planned to seek out more information, whether online or from a medical professional. This reaffirms that health education messages can educate women about the risks of drinking alcohol, as evidenced by earlier studies [12,28,29]. Future research could examine health education messages that are tailored for bisexual and lesbian women and target women who are only light or moderate drinkers.

## 6. Limitations

As Qualtrics handled the distribution of the survey, we could not assert that our sample was a representative sample of U.S. adult women. To mitigate this limitation, we improved external validity by providing quotas for age, race, and ethnicity. However, women who chose to respond to an online survey presumably differ from women who did not. For example, women who responded to this survey may be more concerned about their health than women who chose not to respond; thus, the survey participants may have had a stronger reaction to the health educational messages. Regarding the questions on alcohol screening and counseling in primary care settings, women were asked to recall primary care appointments from the prior two years, introducing the possibility of recall bias. Furthermore, the COVID-19 pandemic could have disrupted access to routine primary care appointments during the time of recall. In addition, our research likely did not account for all confounders, such as employment status, the presence of children, and other types of substance use, which may impact the association among sexual orientation, alcohol use, self-reporting of primary care screening and counseling on alcohol use, and reactions to the health education messages. We excluded employment status as a variable in this analysis since it was highly correlated with income and education level in our data, but it could be useful to consider this in the future. Lastly, bisexual and lesbian women comprised only a small subset of the sample. Future research should consider sexual orientations other than bisexual and lesbian. 

## 7. Conclusions

Our study results align with previous findings that bisexual and lesbian women engage in riskier drinking than heterosexual women. Yet, bisexual and lesbian women were no more likely to be offered advice about harmful alcohol consumption or advised to reduce or quit drinking in a primary care setting than heterosexual women. Although this represents a key opportunity to educate bisexual and lesbian women about the risks of drinking, care must be taken to properly train primary care professionals on the risks of drinking and the unique considerations of bisexual and lesbian women. Bisexual, lesbian, and heterosexual women had similar reactions to messages highlighting that alcohol is a risk factor for breast cancer. Our findings also suggest that women across all three sexual orientations who are harmful drinkers more often agreed to search for more information online or talk to a medical professional than women who are not harmful drinkers. Future research should examine messages tailored to bisexual and lesbian women, as well as differences in messaging for women who engage in harmful drinking compared to those who do not.

## Figures and Tables

**Table 1:** Sociodemographic characteristics, Alcohol Use Disorders Identification Test (AUDIT) scores and categories, and family history of alcohol/drug use by sexual orientation from the ABLE Survey (N = 4891).

Variable (Total N = 4891)	Bisexual N = 554	Lesbian N = 157	Heterosexual N = 4180	Chi-Square, df, *p*-Value
Age				
18–25	280 (50.54%)	60 (38.22%)	688 (16.46%)	504.95, 8, *p* < 0.0001 *
26–35	155 (27.98%)	35 (22.29%)	796 (19.04%)	
36–45	78 (14.08%)	23 (14.65%)	882 (21.10%)	
45–55	34 (6.14%)	24 (15.29%)	876 (20.96%)	
56+	7 (1.26%)	15 (9.55%)	938 (22.44%)	
Race/Ethnicity				
White, Non-Hispanic	413 (74.55%)	99 (63.06%)	2887 (69.07%)	27.61, 10, *p* = 0.002 *
Black/African American, Non-Hispanic	47 (8.48%)	27 (17.20%)	568 (13.59%)	
Hispanic/Latino	72 (13%)	21 (13.38%)	516 (12.34%)	
Asian	8 (1.44%)	0 (0%)	76 (1.82%)	
American Indian/Alaska Native/Native Hawaiian/Pacific Islander	8 (1.44%)	5 (3.18%)	46 (1.10%)	
Other	6 (1.08%)	5 (3.18%)	87 (2.08%)	
Health Insurance Status				
Medicare or Medicaid	262 (47.29%)	62 (39.74%)	1967 (47.11%)	7.35, 4, *p* = 0.12
Private insurance	204 (36.82%)	69 (44.23%)	1662 (39.81%)	
No health insurance	88 (15.88%)	25 (16.03%)	546 (13.08%)	
Missing	0	1	5	
Marital Status				
Married/living with partner	248 (44.77%)	69 (43.95%)	2158 (51.63%)	12.09, 2, *p* = 0.002 *
Single/divorced/widowed	306 (55.23%)	88 (56.05%)	2022 (48.37%)	
Education				
Did not graduate high school	50 (9.09%)	11 (7.01%)	230 (5.52%)	49.95, 4, *p* < 0.0001 *
Graduated high school	429 (78.00%)	114 (72.61%)	2868 (68.89%)	
Bachelor’s degree	71 (12.91%)	32 (20.38%)	1065 (25.58%)	
Missing	4	0	17	
Income				
Yearly income $0 to <$25K	279 (53.55%)	77 (52.03%)	1490 (38.27%)	57.59, 4, *p* < 0.0001 *
Yearly income $25K to <$50K	138 (26.49%)	34 (22.97%)	1178 (30.26%)	
Yearly income $50K+	104 (19.96%)	37 (25.00%)	1225 (31.47%)	
Missing	33	9	287	
Harmful drinking				
Abstainer/low-risk Consumption	404 (72.92%)	110 (70.06%)	3515 (84.09%)	58.95, 2, *p* < 0.0001 *
Harmful drinking	150 (27.08%)	47 (29.94%)	665 (15.91%)	
Family history of drug/alcohol use disorder				
Yes	358 (64.86%)	93 (59.24%)	2018 (48.47%)	57.07, 2, *p* < 0.0001 *
No	194 (35.14%)	64 (40.76%)	2145 (51.53%)	
Missing	2	0	17	

* Statistically significant *p* < 0.05.

**Table 2:** Logistic regression results for harmful drinking outcome from the ABLE Survey (N = 4851).

Variable	Unadjusted Odds Ratio (95% CI)	Adjusted Odds Ratio (95% CI) (N = 4851) ^a^
Sexual Orientation		
Heterosexual	1.00	1.00
Bisexual	1.96 (1.60–2.41) *	1.26 (1.01–1.57) *
Lesbian	2.26 (1.59–3.21) *	1.78 (1.24–2.57) *
Age		
18–25	0.97 (0.79–1.18)	0.95 (0.78–1.17)
26–35	1.00	1.00
36–45	0.70 (0.57–0.86) *	0.74 (0.60–0.92) *
45–55	0.33 (0.26–0.43) *	0.36 (0.28–0.47) *
56+	0.14 (0.10–0.19) *	0.15 (0.11–0.21) *
Race/ethnicity		
White, Non-Hispanic	1.00	1.00
Black/African American, Non-Hispanic	1.24 (1.00–1.53)	1.42 (1.13–1.78) *
Hispanic/Latino	1.30 (1.05–1.61) *	1.15 (0.92–1.45)
Asian	1.36 (0.80–2.30)	1.33 (0.75–2.36)
American Indian/Alaska Native/Native Hawaiian/Pacific Islander	1.14 (0.59–2.21)	1.18 (0.59–2.36)
Other	0.57 (0.29–1.09)	0.58 (0.27–1.23)
Education		
Did not graduate high school	1.05 (0.74–1.49)	0.74 (0.51–1.06)
Graduated high school	1.18 (0.99–1.42)	0.97 (0.80–1.17)
Bachelor’s degree	1.00	1.00
Family history of drug/alcohol use disorder		
No	1.00	1.00
Yes	1.40 (1.21–1.63) *	1.34 (1.15–1.57) *
Marital Status		
Single/divorced/widowed	0.93 (0.80–1.08)	0.91 (0.78–1.06)
Married/living with partner	1.00	1.00

* Significant at *p* < 0.05. ^a^ Adjusted for all other variables listed in this table.

**Table 3:** Alcohol screening and brief counseling in primary care setting questions by sexual orientation from the ABLE Survey (N = 4891).

	Bisexual N = 554	Lesbian N = 157	Heterosexual N = 4180	Chi-Square, df, *p*-Value
Question 1: About how long has it been since you last visited a doctor for a routine checkup?				
Within the past year	311 (56.14%)	85 (54.14%)	2653 (63.53%)	28.71, 10, *p* = 0.001 *
Within the past 2 years	106 (19.13%)	30 (19.11%)	620 (14.85%)	
Within the past 5 years	59 (10.65%)	16 (10.19%)	288 (6.90%)	
5 or more years ago	37 (6.68%)	7 (4.46%)	248 (5.94%)	
Do not know/not sure	22 (3.97%)	10 (6.37%)	208 (4.98%)	
Never	19 (3.43%)	9 (5.73%)	159 (3.81%)	
Missing ^a^	0	0	4	
Question 2: At that checkup, were you asked in person or on a form if you drink alcohol? (within the past year or 2 years to question 1)				
Yes	296 (70.98%)	66 (57.39%)	2195 (67.23%)	8.78, 4, *p* = 0.07
No	91 (21.82%)	35 (30.43%)	822 (25.18%)	
Do not know/not sure	30 (7.19%)	14 (12.17%)	248 (7.60%)	
Missing ^a^	137	42	915	
Question 3: Did the health care provider ask you in person or on a form how much you drink? (affirmative to question 2)				
Yes	200 (67.57%)	48 (72.73%)	1574 (72.07%)	4.02, 4, *p* = 0.402
No	74 (25.00%)	12 (18.18%)	478 (21.89%)	
Do not know/not sure	22 (7.43%)	6 (9.09%)	132 (6.04%)	
Missing ^a^	258	91	1996	
Question 4: Did the health care provider specifically ask whether you drank four or more alcoholic drinks on an occasion? (affirmative to question 3)				
Yes	96 (32.43%)	23 (34.85%)	697 (31.84%)	0.78, 4, *p* = 0.94
No	158 (53.38%)	32 (48.48%)	1177 (53.77%)	
Do not know/not sure	42 (14.19%)	11 (16.67%)	315 (14.39%)	
Missing ^a^	258	91	1991	
Question 5: Were you offered advice about what level of drinking is harmful or risky for your health? (within the past year or 2 years to question 1)				
Yes	64 (15.42%)	16 (13.91%)	440 (13.48%)	11.18, 4, *p* = 0.02 *
No	304 (73.25%)	92 (80.00%)	2587 (79.28%)	
Do not know/not sure	47 (11.33%)	7 (6.09%)	236 (7.23%)	
Missing ^a^	139	42	917	
Question 6: Were you advised to reduce or quit your drinking? (within the past year or 2 years to question 1)				
Yes	44 (10.55%)	15 (13.16%)	298 (9.11%)	6.36, 4, *p* = 0.17
No	350 (83.93%)	91 (79.82%)	2833 (86.61%)	
Do not know/not sure	23 (5.52%)	8 (7.02%)	140 (4.28%)	
Missing ^a^	137	43	909	

* Significant at *p* < 0.05. ^a^ Note that missing values were excluded from the Chi-square analysis.

**Table 4:** Logistic regression results for alcohol screening and brief counseling in primary care setting questions from the ABLE Survey (sample size N specified for each model in the table).

	Doctor Visit for Routine Checkup in the Last Two Years ^a^	Asked in Person or on a Form if You Drink Alcohol ^b^	Asked in Person or on Form How Much You Drink ^c^	Asked about Binge Drinking Specifically ^d^	Offered Advice about Harmful Drinking ^e^	Advised to Reduce or Quit Your Drinking ^f^
	Unadjusted Odds Ratios					
Sexual Orientation ^g^	N = 4647	N = 3505	N = 2386	N = 2183	N = 3503	N = 3631
Heterosexual	1.00	1.00	1.00	1.00	1.00	1.00
Bisexual	0.77 (0.62–0.96) *	1.22 (0.95–1.56)	0.82 (0.62–1.09)	1.03 (0.78–1.35)	1.24 (0.93–1.65)	1.20 (0.86–1.67)
Lesbian	0.76 (0.51–1.14)	0.71 (0.47–1.07)	1.22 (0.64–2.31)	1.21 (0.71–2.09)	1.02 (0.60–1.76)	1.57 (0.90–2.74)
	Adjusted Odds Ratios ^h^					
Sexual Orientation ^g^	N = 4604	N = 3482	N = 2376	N = 2172	N = 3475	N = 3602
Heterosexual	1.00	1.00	1.00	1.00	1.00	1.00
Bisexual	0.96 (0.74–1.23)	1.08 (0.83–1.42)	0.98 (0.72–1.34)	0.88 (0.65–1.18)	0.78 (0.57–1.07)	0.69 (0.48–1.01)
Lesbian	0.84 (0.55–1.29)	0.63 (0.41–0.97) *	1.19 (0.62–2.31)	1.03 (0.58–1.82)	0.70 (0.40–1.24)	1.06 (0.58–1.95)
	Unadjusted Odds Ratios					
Harmful drinking	N = 4647	N = 3505	N = 2386	N = 2183	N = 3503	N = 3631
Abstainers/Low-risk drinkers	1.00	1.00	1.00	1.00	1.00	1.00
Harmful drinkers	0.72 (0.60–0.87) *	1.14 (0.93–1.39)	1.33 (1.02–1.72) *	2.67 (2.15–3.33) *	4.23 (3.44–5.21) *	8.31 (6.59–10.49) *
	Adjusted Odds Ratios ^i^					
Harmful drinking	N = 4604	N = 3482	N = 2376	N = 2172	N = 3475	N = 3602
Abstainers/Low-risk drinkers	1.00	1.00	1.00	1.00	1.00	1.00
Harmful drinkers	0.81 (0.66–0.99) *	0.98 (0.79–1.21)	1.38 (1.05–1.82) *	2.42 (1.92–3.06) *	3.46 (2.78–4.30) *	6.99 (5.47–8.93) *

* Statistically significant *p* < 0.05. ^a^ About how long has it been since you last visited a doctor for a routine checkup? A routine check-up is a general physical exam, not an exam for a specific injury, illness, or condition. ^b^ At that checkup, were you asked in person or on a form if you drink alcohol? ^c^ Did the health care provider ask you in person or on a form how much you drink? ^d^ Did the health care provider specifically ask whether you drank four or more alcoholic drinks on an occasion? ^e^ Were you offered advice about what level of drinking is harmful or risky for your health? ^f^ Health care providers may also advise patients to drink less for various reasons. At your last routine checkup, were you advised to reduce or quit your drinking? ^g^ Respondents who indicated “don’t know/not sure” are excluded from this analysis ^h^ Adjusted for age, race/ethnicity, education, harmful drinking, health insurance status, family history of substance use disorder. ^i^ Adjusted for age, race/ethnicity, education, sexual orientation, health insurance status, and family history of substance use disorder.

**Table 5:** Percentage of respondents who believe that alcohol increases breast cancer risk before and after the brief educational messages from the ABLE Survey (N = 4891).

Variable (N = 4891)	Harmful Drinking = No				Harmful Drinking = Yes			
	Bisexual	Lesbian	Heterosexual	Chi-Square, df, *p*-Value	Bisexual	Lesbian	Heterosexual	Chi-Square, df, *p*-Value
Belief that alcohol increases breast cancer risk (before the educational message)								
Yes	105 (26.45%)	32 (29.63%)	812 (23.33%)	6.77, 4, *p* = 0.15	42 (28.19%)	7 (15.56%)	193 (29.29%)	4.62, 4, *p* = 0.33
No	118 (29.72%)	38 (35.19%)	1192 (34.25%)		61 (40.94%)	24 (53.33%)	264 (40.06%)	
Do not know	174 (43.83%)	38 (35.19%)	1476 (42.41%)		46 (30.87%)	14 (31.11%)	202 (30.65%)	
Missing ^a^	7	2	35		1	2	6	
Belief that alcohol increases breast cancer risk (after the educational message)								
Yes	202 (50.25%)	62 (56.88%)	1788 (51.35%)	8.25, 4, *p* = 0.08	75 (50.68%)	20 (44.44%)	326 (49.92%)	1.83, 4, *p* = 0.77
No	107 (26.62%)	28 (25.69%)	780 (22.40%)		41 (27.70%)	17 (37.78%)	188 (28.79%)	
Do not know	93 (23.13%)	19 (17.43%)	914 (26.25%)		32 (21.62%)	8 (17.78%)	139 (21.29%)	
Missing ^a^	2	1	33		2	2	12	
Difference in belief = yes pre/post	+23.80%	+27.25%	+28.02%		+22.49%	+28.88%	+20.63%	

^a^ Note that missing values were excluded from the analysis.

**Table 6:** Percentage of respondents who agree with action intentions post-survey intervention from the ABLE Survey (N = 4891).

Variable (N = 4891)	Harmful Drinking = No					Harmful Drinking = Yes				
	Bisexual	Lesbian	Heterosexual	Chi-Square, df, *p*-Value	Bisexual, Lesbian, and Heterosexual Women	Bisexual	Lesbian	Heterosexual	Chi-Square, df, *p*-Value	Bisexual, Lesbian, and Heterosexual Women
Search for more information online about the link between alcohol and breast cancer										
Agree	206 (51.12%)	48 (43.64%)	1517 (43.38%)	10.62, 4, *p* = 0.03 *	1771 (44.16%)	93 (62.00%)	29 (63.04%)	428 (64.56%)	1.80, 4, *p* = 0.77	550 (64.03%)
Neither agree/disagree	135 (33.50%)	38 (34.55%)	1372 (39.23%)		1545 (38.53%)	43 (28.67%)	13 (28.26%)	161 (24.28%)		217 (25.26%)
Disagree	62 (15.38%)	24 (21.82%)	608 (17.39%)		694 (17.31%)	14 (9.33%)	4 (8.70%)	74 (11.16%)		92 (10.71%)
Missing	1	0	18		19	0	1	2	3	
Talk to a medical professional about the link between alcohol and breast cancer										
Agree	147 (36.57%)	43 (39.09%)	1139 (32.73%)	6.03, 4, *p* = 0.20	1329 (33.29%)	70 (46.67%)	24 (52.17%)	345 (51.96%)	3.47, 4, *p* = 0.48	439 (51.05%)
Neither agree/disagree	156 (38.81%)	36 (32.73%)	1458 (41.90%)		1650 (41.33)	49 (32.67%)	17 (39.96%)	211 (31.78%)		277 (32.21%)
Disagree	99 (24.63%)	31 (28.81%)	883 (25.37%)		1013 (25.38%)	31 (20.67%)	5 (10.87%)	108 (16.27%)		144 (16.74%)
Missing	2	0	35		37	0	1	1		2

* Statistically significant *p* < 0.05.

## Data Availability

The dataset analyzed in the current study is available upon reasonable request to the corresponding author.
